# Young fibroblast-derived migrasomes alleviate keratinocyte senescence and enhance wound healing in aged skin

**DOI:** 10.1186/s12951-025-03293-2

**Published:** 2025-03-11

**Authors:** Hanlin Tu, Yingliang Shi, Yi Guo, Zhongyang Zou, Yuyan He, Jing Zhou, Sangang He, Guoliang Sa

**Affiliations:** 1 State Key Laboratory of Oral & Maxillofacial Reconstruction and Regeneration, Key Laboratory of Oral Biomedicine Ministry of Education, Hubei Key Laboratory of Stomatology, School & Hospital of Stomatology, Wuhan University, Wuhan, 430079 China; 2 Department of Oral and Maxillofacial surgery, School and Hospital of Stomatology, Wuhan University, Wuhan, 430079 China

**Keywords:** Migrasomes, Fibroblast, Senescence, Skin wound healing, Aging

## Abstract

**Background:**

Alterations in intercellular communication driven by cellular senescence constitute an important factor in skin aging. Migrasome, a newly discovered vesicular organelle, efficiently participates in intercellular communication; however, the relationship between cellular senescence and migrasomes remains unreported.

**Objective:**

This study aims to explore the possible relationship between cellular senescence and migrasomes formation, and investigate the effects of young fibroblast-derived migrasomes on senescent keratinocytes and wound healing in aged skin.

**Result:**

Single-cell RNA sequencing (scRNA-seq) data analysis revealed that fibroblasts exhibited the highest level of transcriptional variability during skin aging, and the degree of fibroblast senescence negatively correlated with the expression level of migrasome-associated markers. Further multiplex Immunohistochemistry (mIHC) results suggested that younger mouse skin contained more migrasomes than older mouse skin. Transmission electron microscopy (TEM) observations demonstrated abundant migrasomes in the skin from young individuals. In vitro experiments indicated that young fibroblasts produced significantly more migrasomes than senescent fibroblasts, as confirmed by wheat germ agglutinin (WGA) staining and scanning electron microscopy (SEM). Importantly, purified migrasomes from young fibroblasts were found to reduce the expression of senescence-associated markers in HaCaT cells. In vivo, using a wound healing model in naturally aged mice, we observed that migrasomes derived from young fibroblasts not only accelerated wound healing but also reduced senescence-associated marker expression in the skin.

**Conclusion:**

Migrasomes formation ability reduced during skin aging progress, and young fibroblast-derived migrasomes rejuvenated senescent keratinocytes and promoted wound healing in aged skin. These findings offer new ideas for alleviating skin aging and enhancing wound healing in aged skin.

**Graphical abstract:**

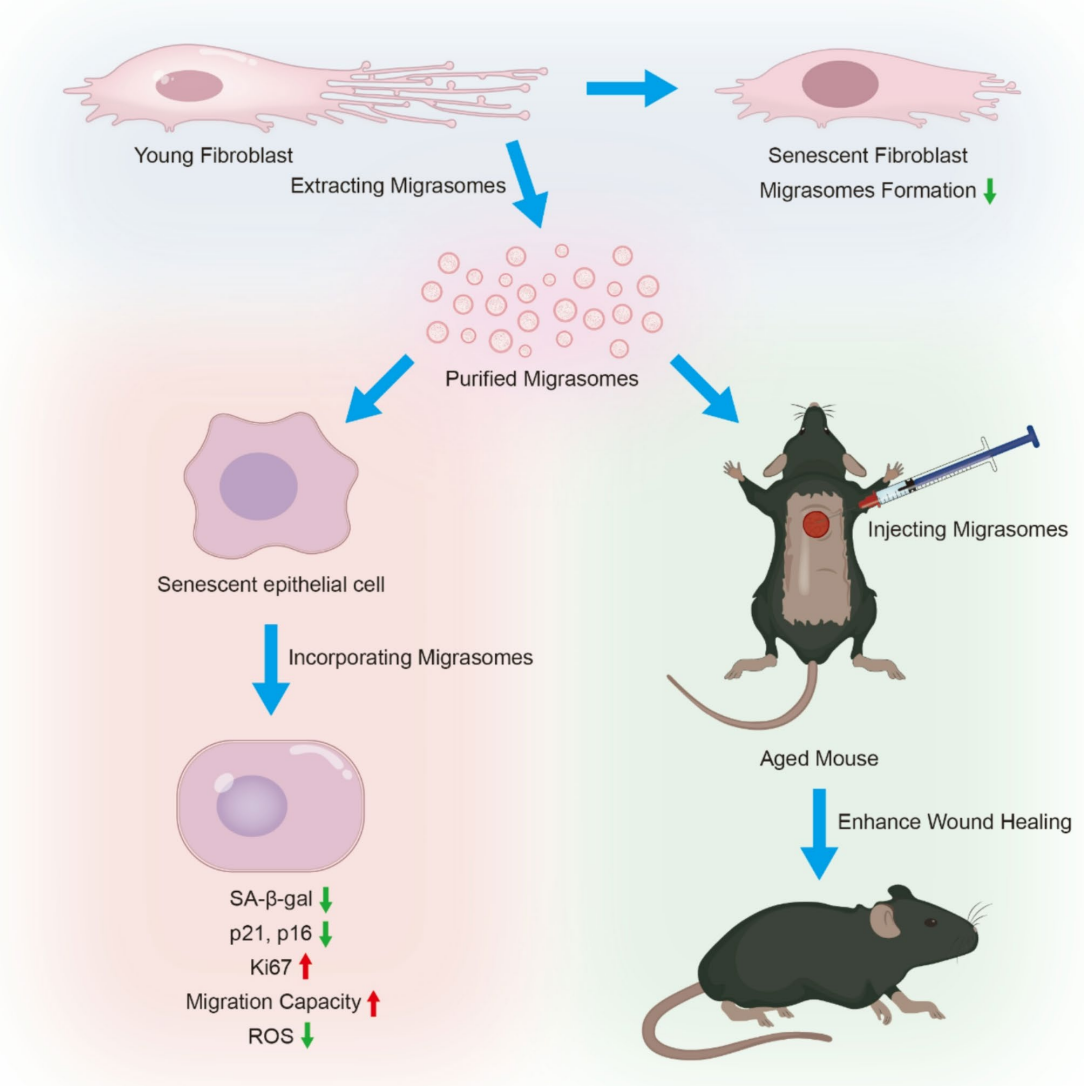

**Supplementary Information:**

The online version contains supplementary material available at 10.1186/s12951-025-03293-2.

## Introduction

As individuals age, the skin inevitably undergoes a transition from a youthful state to senescence, which results in a progressive decline in its barrier function and regenerative capabilities [1]. Furthermore, skin aging is associated with delayed wound healing, and an increased susceptibility to various skin diseases [2].

Aging is one of the major risk factors for wound complications and chronic wound healing [3]. The persistence and accumulation of senescent cells are significant contributors to skin aging. These cells not only closely relate to aging but also impact the repair and regeneration potential of damaged tissues [4]. Cell senescence is a state of irreversible cell cycle arrest triggered by stress-induced damage or certain physiological processes. A key feature of senescent cells is the upregulation of cyclin-dependent kinase (CDK) inhibitors, p21 and p16. These proteins inhibit CDK-cyclin complexes, leading to cell cycle arrest at the G1 phase [5]. During this process, cells typically aberrantly secrete a variety of pro-inflammatory cytokines, chemokines, and matrix metalloproteinases, collectively referred to as the senescence-associated secretory phenotype (SASP) [6]. The persistent release of SASP may contribute to chronic inflammation, fueling wounds that fail to heal [7]. Eliminating senescent cells is considered an effective strategy to mitigate the aging process [8]. Clearance of p16 has been shown to delay age-related diseases [8]. Additionally, clearance of p21-highly expressing senescent cells accelerates skin wound healing, partly mediated by NF-κB inhibition [9].

The interaction between epithelial cells and mesenchymal cells has increasingly been recognized as a crucial factor in biological processes such as wound healing and tissue regeneration [10, 11]. Mesenchymal cells also play a significant role in regulating epithelial cell senescence. For example, the deficiency of SLC3A2 in dermal fibroblasts can result in premature senescence and loss of homeostasis in epithelial cells [12]. Autologous fat grafting has been shown to restore normal epidermal matrix and reverse aging [13]. Currently, it is widely believed that their effects mainly rely on the paracrine system, which includes various bioactive factors such as growth factors, cytokines, and extracellular vesicles. Notably, skin aging is accompanied by a gradual decline of mesenchymal cell function, which leads to alteration in intercellular communication between epithelial cells and mesenchymal cells [14].

Recently, a unique vesicular organelle migrasome has been discovered within biological organisms [15]. These vesicular structures originate from retraction fibers at the rear of migrating cells, growing to sizes of 0.5 to 3 μm. Tetraspanin 4 (TSPAN4) has been found to play an important role in their formation [16]. Migrasomes are involved in many biological processes, including intercellular communication, material exchange, the maintenance of homeostasis, and embryonic development [17]. However, the relationship between cellular senescence and migrasomes has remained unreported. This study aims to explore the relationship between migrasomes and cellular senescence. Additionally, we will investigate the impact of young fibroblast-derived migrasomes on keratinocyte senescence and on wound healing in aged skin.

## Materials and methods

### Ethics statement

#### Ethical approval

Ethical approval for human sample collection was obtained from the Ethics Committee of School & Hospital of Stomatology, Wuhan University. All animal experiments were conducted at the Center for Animal Experiment/Animal Biosafety Level-III laboratory of Wuhan University.

### Downloading and processing raw data of scRNA-seq

We downloaded single-cell RNA sequencing (scRNA-seq) data related to aging skin (GSE130973) from the Gene Expression Omnibus (GEO). The sequencing data included five skin samples obtained from two ‘young’ (25 and 27 years old) and three ‘old’ (53, 69, and 70 years old) male Caucasian donors with a defined, sun-protected area. Using the Cell Ranger software suite (v7.2.0, 10x Genomics) with the default parameters, we evaluated the quality of the sample-specific FASTQ files. The reads were aligned to the human reference genome (GRCh38) using a STAR aligner to generate a digital gene expression matrix, with expression levels determined by unique molecular identifiers (UMIs). The filtered gene expression matrices were then prepared for downstream analyses.

### ScRNA-seq data analysis and cell-type identification

We used the Seurat R package (version 4.4.0) to further analyze the scRNA-seq data [18]. After assessing Cell Ranger metrics, we excluded cells with fewer than 200 genes, more than 7,500 genes, or over 5% mitochondrial genes, leaving 25,031 cells for further analysis.

To address inter-individual differences and batch effects, we integrated all samples using Seurat’s standard protocol. First, we normalized UMI counts with the “LogNormalize” method, transforming the data via natural logarithm. We identified the 2,000 most variable genes per sample using the FindVariableFeatures() function. Next, FindIntegrationAnchors() was applied to identify integration anchors across the datasets, followed by integration using IntegrateData(), both functions using 30 canonical correlation analysis (CCA) dimensions.

The integrated data underwent standard clustering and visualization with Seurat, starting with scaling via ScaleData() and principal component analysis (PCA) using RunPCA(). Unsupervised clustering was performed using FindNeighbors() and FindClusters(). To determine the optimal PCA dimensions for FindNeighbors(), we employed the JackStraw function, which compares null distribution-based PCA scores. We settled on the first 20 PCA dimensions to construct a Shared Nearest Neighbor (SNN) graph. Clusters were formed with a resolution of 0.6 in the FindClusters() function using a SNN modularity optimization-based clustering algorithm. To determine this appropriate resolution, we set parameter “resolution = seq(from = 0.1, to = 1.0, by = 0.1)” in the FindCluster() function and visualized it by using the clustree() function. Nonlinear dimensionality reduction was performed using the RunTSNE function with 20 PCA dimensions, visualized by t-Distributed Stochastic Neighbor Embedding (t-SNE).

To identify genes enriched in each cell cluster, we used the FindAllMarkers() function with parameters set to “only.pos = TRUE, min.pct = 0.25, logfc.threshold = 0.25”. Significant marker genes were defined as those with|‘avg_logFC’| > 0.25 and ‘p_val_adj’ < 0.05. These genes, along with literature-derived markers, were used to establish cell identities, with average expression projected onto t-SNE or violin plots. Clusters not fitting known cell types were categorized as “Other”. Significant marker genes for each cluster are detailed in Table S1.

For secondary clustering of fibroblasts, we extracted the “Fibroblasts” cluster and ran the previous steps again, using a resolution of 0.1 for the FindClusters() function. For visualization, we recalculated the tSNE plot using the RunTSNE() function with default parameters and 20 PCA dimensions.

### Identification of aging-associated differentially expressed genes (DEGs)

To identify aging-associated DEGs between old and young groups (O/Y) for each cell type, we employed the FindMarkers() function with parameters set to “verbose = FALSE, test.use = ‘wilcox’, min.pct = 0.1.” We considered genes significant if|‘avg_logFC’| > 0.25 and ‘p_val_adj’ < 0.05. Aging-associated DEGs for each cell type are listed in Table S2. 

### Downloading of the aging-database

In order to find out which cell type played the biggest role in senescence, we downloaded four databases of aging-related genes (shown below and listed in the Table S3) and compared the aging-associated DEGs of each cell type with the aging-related genes from the following four databases.

GenAge: https://genomics.senescence.info/genes/index.html [19].

CellAge: http://genomics.senescence.info/cells [20].

CSGene: https://bioinfo-minzhao.org/csgene [21].

Aging Atlas: https://ngdc.cncb.ac.cn/aging/index [22].

### Aging-database score analysis

The method we used for aging-database score analysis is “UCell” (https://github.com/carmonalab/UCell), which is based on the Mann-Whitney U statistic, is robust to dataset size and heterogeneity, and is more efficient than other robust methods in terms of computation time and memory requirements [23]. To use UCell, we used the irGSEA.score(method = “UCell”) function of the irGSEA R package (version 3.3.1) [24], which can batch score and visualize four databases. And we compared it to the AddModuleScore_UCell() function of the UCell R package (version 2.6.2) and the results were exactly the same. Scoring results can be seen in the supplementary information.

### Correlation analysis of migrasome genes and aging-databases

To explore the relationship between aging and migrasomes formation in fibroblasts cluster, we scored the two types of genes and calculated correlations from the scoring results by the function ggscatterstats(type = “spearman”) of the ggstatsplot R package (version 0.12.3) [25].

### Cell culture

The BJ cell was purchased from the National Collection of Authenticated Cell Culture. Primary young fibroblasts were isolated from the oral mucosal tissues of healthy young volunteers (*n* = 5), with their purity confirmed by immunofluorescence staining. Fibroblasts from passages 3–5 were used for subsequent experiments. HaCaT cells, BJ cells, and primary young fibroblasts were cultured in DMEM (C11995500BT, Gibco, USA) supplemented with 10% fetal bovine serum (C04002, VivaCell, Germany) and 1% penicillin-streptomycin (P1400, Solarbio, China) at 37 °C and 5% CO_2_.

### Mouse skin wound model and treatments

All C57BL/6 mice (8 weeks and 64 weeks of age) were purchased from the Hubei Provincial Center for Disease Control and Prevention. Prior to the experimental procedures, the mice were housed under observation for one week. Mice were anesthetized by intraperitoneal injection of 30 µL/g tribromoethanol and full-thickness skin wounds (1 mm in diameter) were made on the back of each mouse after shaving. The mice were randomly assigned to four groups: Young Mouse Group (Y), Aged Mouse Control Group (AC), Aged Mouse PBS-Injected Group (AP), and Aged Mouse Migrasome-Injected Group (AM). The young mouse group consists of 3 mice, and the aging mouse groups consist of 5 mice each. AM is treated with 100 µg migrasomes dissolved in 100 µL PBS (C10010500BT, Gibco, USA) and AP is treated with 100 µL PBS. On days 0, 3, 5, 7 and 12 after injury, the wounds were photographed and measured. On day 12 after injury, mice were euthanized, and skin samples were collected. Half of the samples were used for Western blot analysis, while the other half were fixed in 4% paraformaldehyde (PFA) for subsequent histological examination.

### Isolation of migrasomes from cultured cells

As described in the referenced article, cells were cultured in 150 mm dishes coated with 1 µg/ml fibronectin (F0895, sigma, USA) [26]. Cells and migrasomes were digested with 0.25% trypsin, and collected in 50 ml tubes. All subsequent steps were performed at 4 °C. After centrifuging at 1000 g for 10 min, the supernatant was further centrifuged at 4000 g for 20 min, and then at 20,000 g for 30 min to collect the pellet. The crude migrasomes pellet was washed twice with PBS. Then the crude migrasomes were performed by density gradient centrifugation using Optiprep kit (LYSISO1, Sigma-Aldrich, USA). In a SW32Ti rotor (Beckman) at 4 °C, centrifuge at 150,000 g for 4 h. Each fraction was mixed with an equal volume of PBS (1 ml) and centrifuged at 20,000 g for 30 min, and the pellet was collected. After two washes with PBS, migrasome concentration was measured with the BCA Protein Assay Kit (P0010S, Beyotime, China) and the migrasomes were stored at -80 °C for further experiments.

### Statistical analysis

Data are expressed as mean ± standard error of the mean (SEM). GraphPad Prism 9.5 was applied for statistical analysis. One-way ANOVA analysis, Mann-Whitney test, Welch’s t test, Kruskal-Wallis test, Welch’s ANOVA, and Two-way ANOVA analysis were used. Specific statistical analysis is shown in figure legend. Statistically significant differences were considered at * *P* < 0.05, ** *P* < 0.01, and *** *P* < 0.001.

## Results

### Fibroblasts possessed the highest level of transcriptional variability during skin aging

**Fig. 1:**
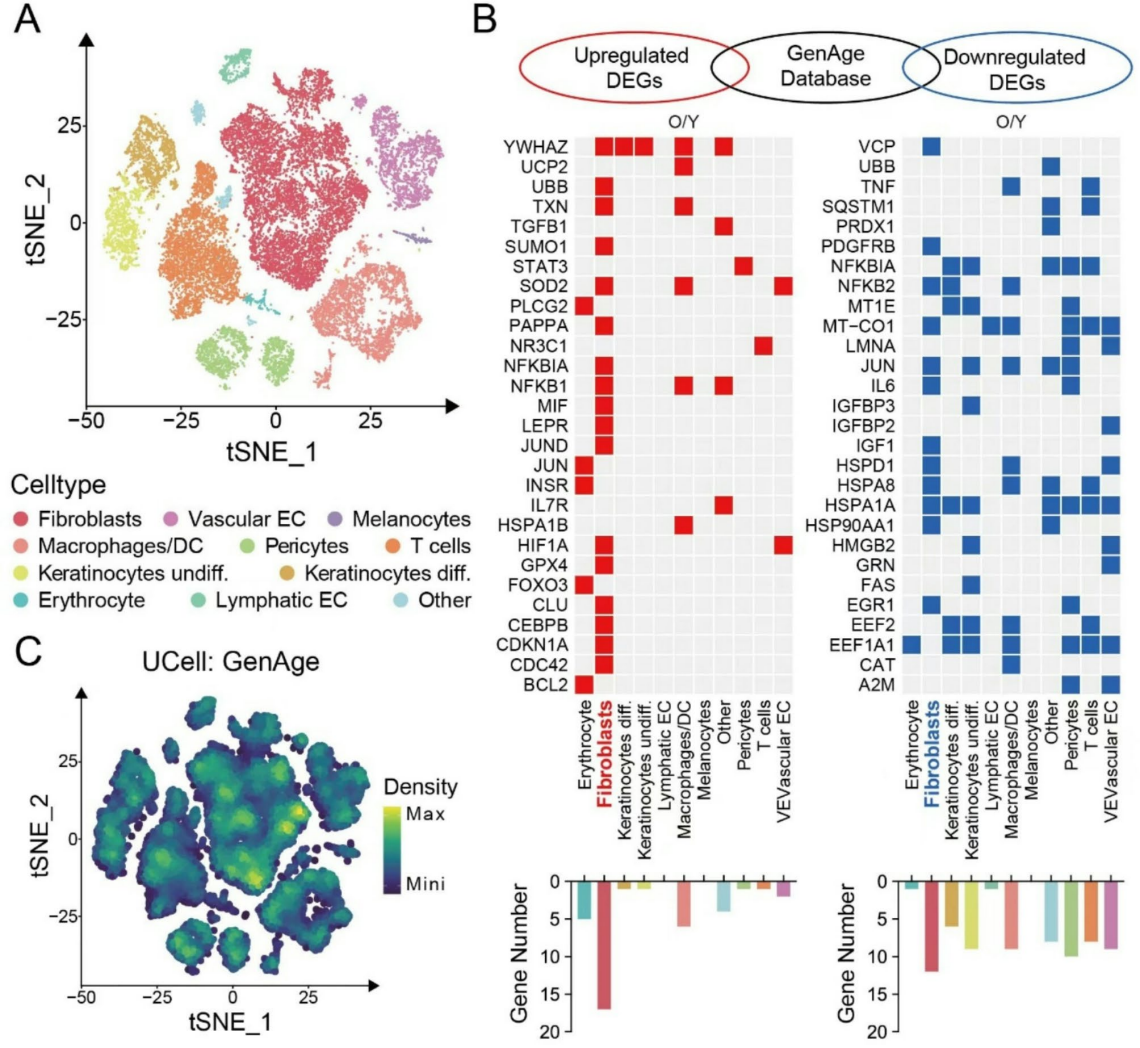
Single-cell RNA sequencing analysis reveals the role of fibroblasts in aging skin **A**. t-Distributed Stochastic Neighbor Embedding (t-SNE) plot depicting single-cell transcriptomes from whole human skin (*n* = 5). Each dot represents a single cell (*n* = 25,031). Cells are colored by types and annotated to the right **B**. Heatmap showing genes shared between aging-related genes in the GenAge database and aging-associated DEGs for each skin cell type. Only the ensembles of shared genes are shown, and the number statistics are shown below the heatmap **C**. Mapping density plots of t-SNE showing scoring results from GenAge databases (see Methods for details). Yellow color indicates maximum gene expression and dark blue color indicates low or no gene expression

To describe the dynamic changes in the proportions and functions of various cell types during skin aging, we analyzed single-cell RNA sequencing (scRNA-seq) data from human samples. This data was sourced from the Gene Expression Omnibus (GEO) database (GSE130973). Eleven distinct skin cell types, including epithelial cells, fibroblasts, endothelial cells, and immune cells, were annotated (Fig. 1A). To further explore the molecular changes underlying human skin aging, we performed a comprehensive comparison analysis of aging-related differentially expressed genes (DEGs) with aging and longevity associated gene from GenAge database [19]. Results showed that fibroblasts possessed the highest level of transcriptional variability among all identified skin cell types during the process of skin aging (Fig. 1B and C). Identical comparative analyses were conducted using three independent databases: Aging Atlas, CSGene, and CellAge [20–22], and the results were consistent with our initial findings (Fig. S1, Fig. S2, and Fig. S3,). Consequently, we focused on fibroblasts in the subsequent analysis.

### ScRNA-seq revealed migrasomes formation negatively related with cellular senescence

**Fig. 2:**
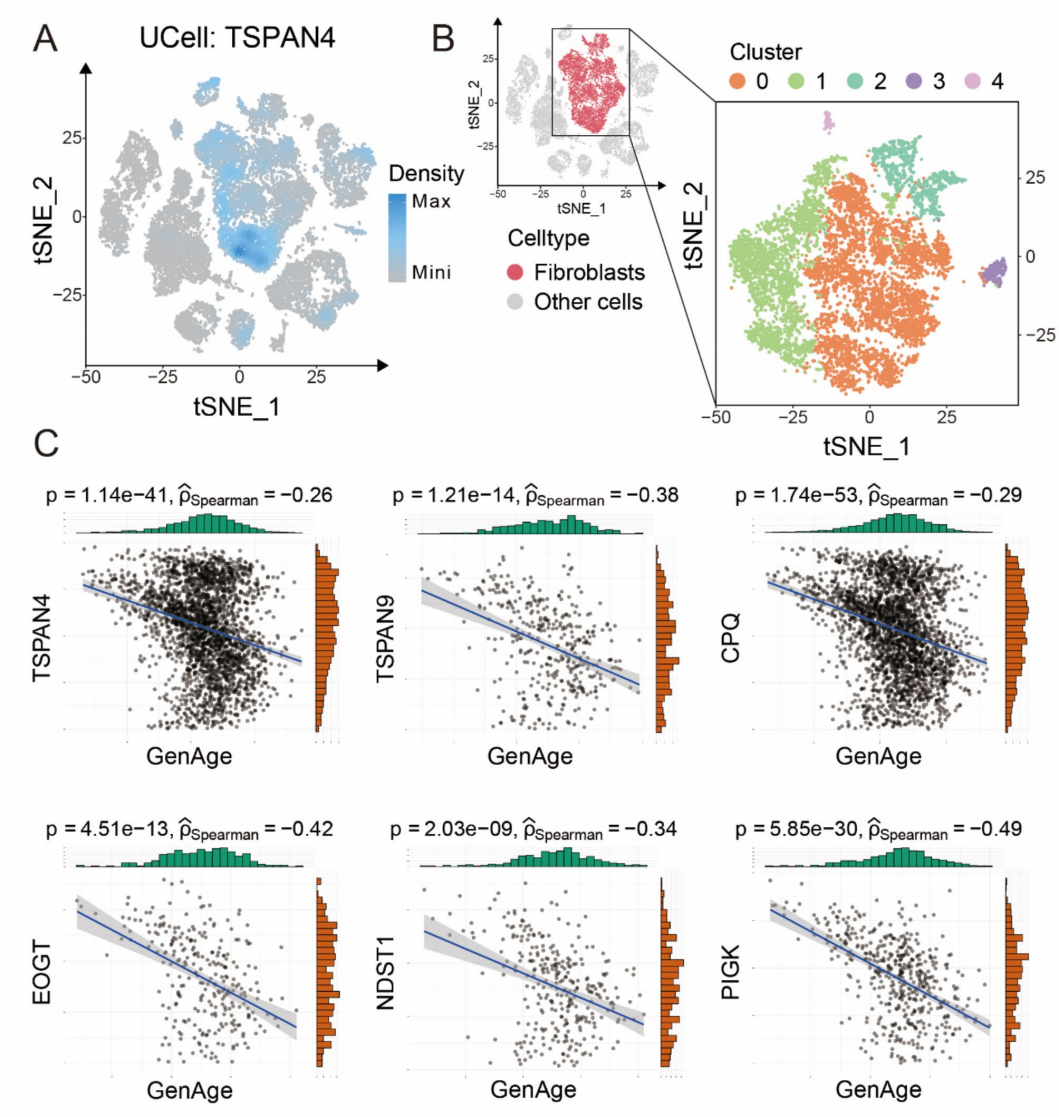
ScRNA-seq suggests a potential relationship between aging and migrasomes formation **A**. Mapping density plot of t-SNE showing the scoring result for the migrasome-associated gene TSPAN4. Dark blue color indicates maximum gene expression and grey color indicates low or no gene expression **B**. t-SNE diagram illustrated the cell clusters of fibroblasts **C**. Scatterplots showing the correlation between the six migrasome-associated genes (TSPAN4, TSPAN9, CPQ, EOGT, NDST1 and PIGK) and aging-related genes in the GenAge database respectively (see Methods for details). The trend lines represent the overall trend of all the scatter points in the corresponding plots, and the corresponding p-value and Spearman’s correlation coefficient are shown at the top of the plots

TSPAN4 was identified as a critical promoter of migrasomes formation, and overexpression of TSPAN4 significantly enhanced the capacity of migrasomes formation [27]. To explore the relationship between migrasomes and aging, we performed enrichment analysis directing to the TSPAN4 gene from Gene Expression Omnibus (GEO) database (GSE130973). Interestingly, the results indicated that TSPAN4 was also predominantly expressed in fibroblasts (Fig. 2A). We extracted the fibroblasts cluster (Fig. 2B), and an independent analysis of fibroblasts demonstrated that the migrasome-related markers TSPAN4, TSPAN9, CPQ, EOGT, PIGK and NDST1 were negatively correlated with aging-related genes (|r| > 0.25) in GenAge database (Fig. 2C). Similarly, we conducted correlation analyses in three additional databases Aging Atlas, CSGene, and CellAge, and the results were consistent to that correlation analyses result conducted with GenAge database (Fig. S4, S5 and S6). These results suggested that the senescent process in fibroblasts might affect the formation of migrasomes.

### Fibroblast senescence reduces the formation of migrasomes

**Fig. 3:**
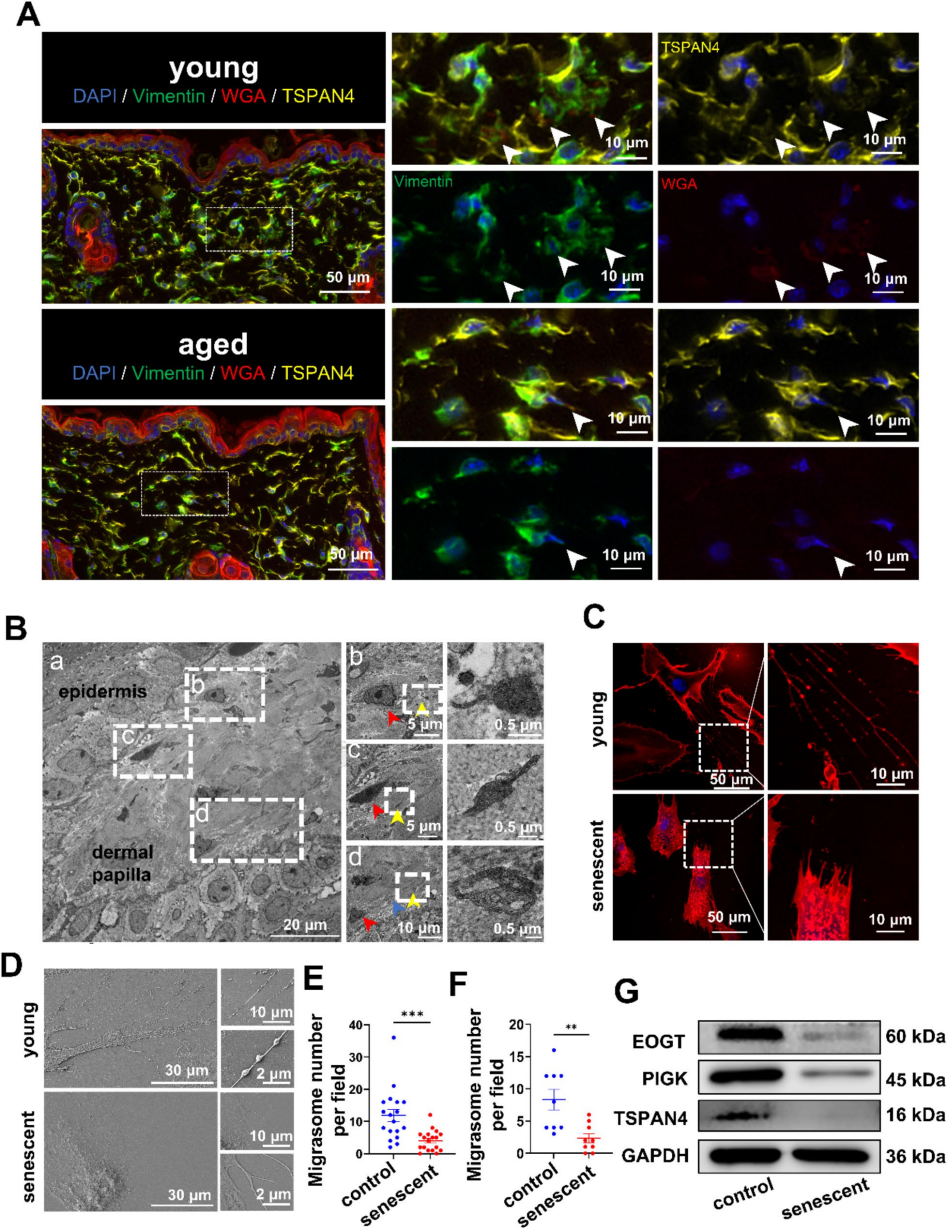
Senescent fibroblasts significantly impair migrasomes formation **A**. mIHC of skin from young and aged mice. Skin sections from three 8-wFeek-old young mice and three 64-week-old aged mice were subjected to vimentin, TSPAN4, and WGA staining. Vimentin (green) identifies fibroblasts, while the colocalization of TSPAN4 (yellow) and WGA (red) denotes migrasomes. White arrows denote colocalization of Vimentin, TSPAN4 and WGA. Representative images are presented **B**. TEM of skin sample from young individuals. (**a**) Shows the dermal papilla structure; (**b**, **c**, **d**) present magnified views of migrasome-like structures. Yellow arrows denote migrasome-like structures, blue arrows indicate retraction fiber-like structures, and red arrows highlight potential migrasome precursor cells **C**. WGA staining images of young BJ cells and H_2_O_2_-induced senescent BJ cells, with magnified views **D**. SEM images of young BJ cells and H_2_O_2_-induced senescent BJ cells, with magnified views **E**. Quantification of migrasome numbers in young BJ cells and H_2_O_2_-induced senescent BJ cells using WGA staining. The experiment was independently repeated three times, with six random fields analyzed per group. Data are presented as mean ± SEM with significance (by normality and lognormality test followed by Mann-Whitney test) **F**. Quantification of migrasome numbers in young BJ cells and H_2_O_2_-induced senescent BJ cells was performed using scanning electron microscopy. The experiment was independently repeated three times, with three random fields analyzed per group. Data are presented as mean ± SEM with significance (by normality and lognormality test followed by Welch’s t test) **G**. Expression levels of migrasome markers in young BJ cells and H₂O₂-induced senescent BJ cells analyzed by Western blot

To further validate the relationship between migrasomes formation in fibroblasts and senescence, we performed multiplex Immunohistochemistry (mIHC) using skin tissues from three young (8 weeks old) and three aged (64 weeks old) C57BL/6J mice. Vimentin was used to label fibroblasts, and the co-localization of TSPAN4 and WGA was used to indicate migrasomes [28, 29]. Young mice skin exhibited a higher co-localization ratio of vimentin, TSPAN4, and WGA, suggesting that young fibroblasts produce more migrasomes (Fig. 3A). To further confirm the presence of migrasomes in skin, we analyzed skin from young individuals using transmission electron microscopy (TEM). We observed a notable accumulation of migrasome-like structures (yellow arrows) in the dermal papillae, characterized by large oval vesicles (0.5–3.0 μm in diameter) (Fig. 3B). These migrasome-like structures contained various smaller vesicles (Fig. 3B, yellow arrow), and were closely associated with retraction fibers (Fig. 3B, blue arrow), aligning with previous literature [15]. Adjacent to the migrasomes, a leading cell (Fig. 3B, red arrow) with a spindle or irregular shape was observed. The cell’s cytoplasm was rich in expanded rough endoplasmic reticulum, which is characteristic of fibroblasts.

To further validate that senescence process in fibroblasts may inhibit the formation of migrasomes, we conducted in vitro experiments. We induced senescence in the BJ cells line using H_2_O_2_, which is a fibroblast cell line established from skin taken from normal foreskin. Senescence-associated β-galactosidase (SA-β-gal) staining showed that the percentage of SA-β-gal positive cells increased in H_2_O_2_-treated BJ cells (Fig. S7A). Western blot revealed an increased expression of the senescence markers p16 and p21 (Fig. S7B). These results demonstrated the successful establishment of a senescent BJ cell model. Wheat germ agglutinin (WGA) staining showed that senescent fibroblasts had increased cell volume and a flattened morphology, which was consistent with cellular senescence. Notably, non-senescent fibroblasts had significantly higher migrasomes formation than senescent fibroblasts (Fig. 3C and E), as confirmed by scanning electron microscopy (Fig. 3D and F). Furthermore, Related markers of migrasome including EOGT, PIGK and TSPAN4 were significantly upregulated in non-senescent fibroblasts (Fig. 3G). These findings collectively support our hypothesis that young fibroblasts have the ability to produce migrasomes more efficiently than senescent fibroblasts.

### Young fibroblast migrasomes alleviate senescence in HaCaT cell in vitro

**Fig. 4:**
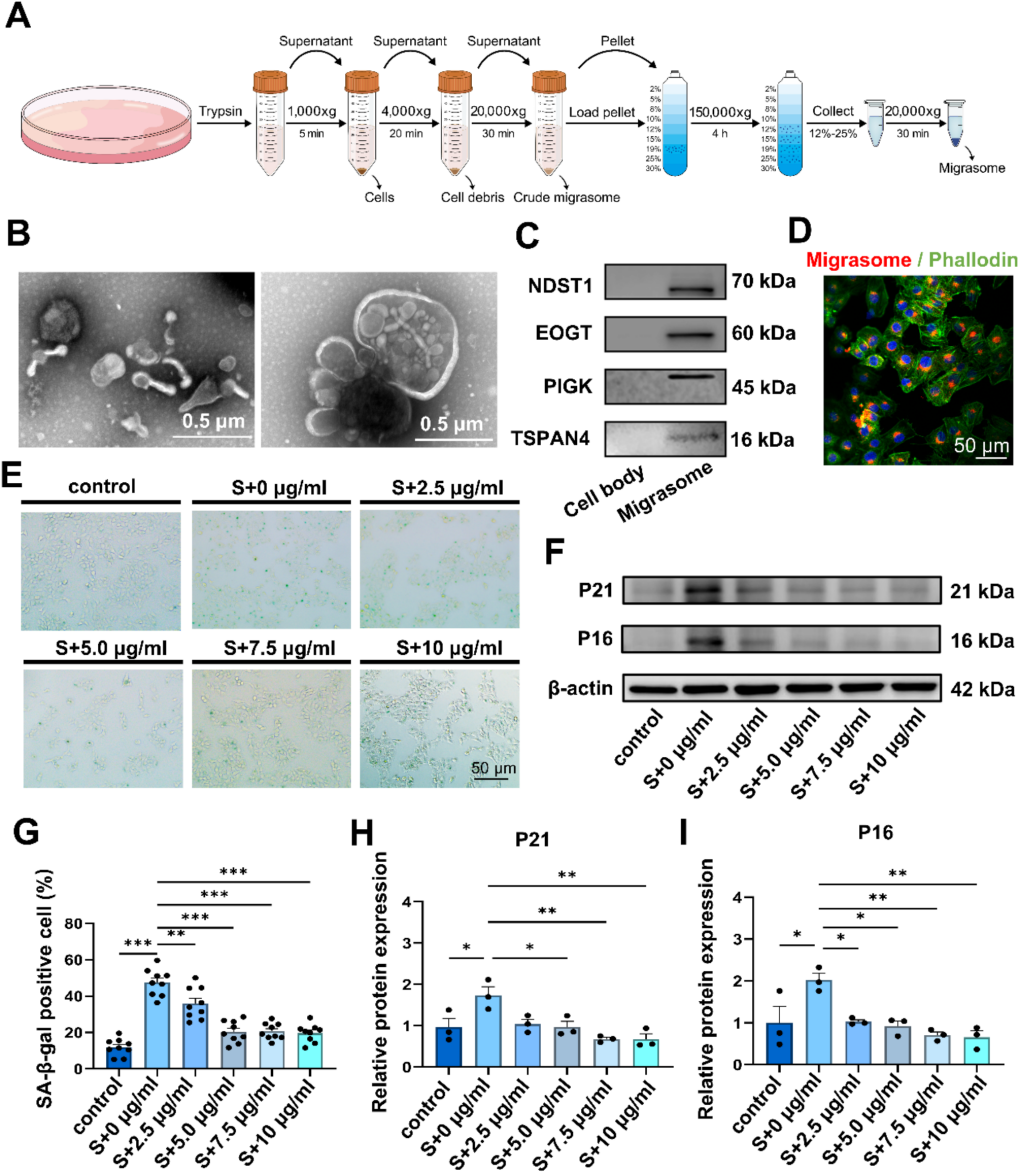
Young fibroblast-derived migrasomes alleviate senescence in HaCaT in vitro **A**. Figure of the purification procedure for young fibroblast-derived migrasomes **B**. Representative TEM images of negative staining for purified young fibroblast-derived migrasomes **C**. Representative images of western blot showing the expression of migrasome-related markers in purified young fibroblast-derived migrasomes **D**. The internalization of migrasomes by senescent HaCaT cells. The migrasomes are specifically labeled with WGA and appear as red puncta, and phalloidin staining in green **E**. Representative images of SA-β-gal staining in H₂O₂-induced senescent HaCaT cells treated with different concentrations of young fibroblast-derived migrasomes **F**. Representative Western Blot images showing the expression of senescence markers p16 and p21 in H₂O₂-induced senescent HaCaT cells treated with different concentrations of young fibroblast-derived migrasomes n **G**. Quantification of SA-β-gal positive cells in H₂O₂-induced senescent HaCaT cells treated with different concentrations of migrasomes for 48 h. Experiments were repeated independently three times. Data were collected by randomly photographing 3 fields per group. Statistical analysis was performed using one-way ANOVA. Error bars indicate the mean ± SEM H and I. Quantitative Western Blot analysis showing the expression of senescence markers p16 and p21 in H₂O₂-induced senescent HaCaT cells after 48 h of stimulation with various concentrations of migrasomes. Experiments were conducted independently three times. Data were analyzed using one-way ANOVA. Error bars indicate the mean ± SEM

Considering that migrasomes are deposited near the epidermal-dermal junction and the key role of fibroblast-epithelial interactions in skin aging, we further study the impact of fibroblast-derived migrasomes on the senescence of the epithelial cell. To this end, we first extracted fibroblasts from young people and identified them by vimentin immunofluorescence staining (Fig. 8A). Then we purified young fibroblast-derived migrasomes via density gradient centrifugation. Purified migrasomes exhibit a typical pomegranate-like structure with visible interconnected retraction fibers structures, and have a diameter of approximately 0.5–3 μm (Fig. 4B). Migrasome-related markers were highly expressed in the purified sample (Fig. 4C). These characteristics were consistent with the migrasomes reported previously [15].

It has been reported that H₂O₂ can induce senescence in HaCaT cells, a typical keratinocyte line [30]. Therefore, we established a senescence model using HaCaT cells induced by H₂O₂ at various concentrations (200, 400, 600, 800, and 1000 µmol/L) for 4 h. Subsequently, 48 h after induction, the senescent HaCaT cells were identified using several well-recognized senescence biomarkers. SA-β-gal staining demonstrated that H₂O₂-treated HaCaT cells exhibited an enlarged and flattened morphology, and an increased percentage of SA-β-gal-positive cells was observed (Fig. S8B and S8D). Western blot analysis further revealed that H₂O₂ treatment significantly upregulated the expression of senescence markers p16 and p21 (Fig. S8C, S8E, and S8F). These results indicated that the degree of senescence in HaCaT cells seemed to correlate with the H₂O₂ concentration. However, when the concentration exceeded 600 µmol/L, the number of adherent cells decreased. Consequently, we selected 600 µmol/L H₂O₂ for subsequent experiments. To investigate whether migrasomes can be internalized by senescent HaCaT cells, we introduced WGA-labeled migrasomes into senescent HaCaT cells. The results revealed that the migrasomes were dispersed within the cells (Fig. 4D). We then treated the senescent HaCaT cells with different concentrations of fibroblast-derived migrasomes (2.5, 5, 7.5 and 10 µg/ml). Young fibroblast-derived migrasomes reduced the percentage of SA-β-gal positive cells (Fig. 4E and G). Western blot showed that migrasomes significantly reduced the H₂O₂-induced elevation of p16 and p21 (Fig. 4F, H and I). According to these results, we found that the optimal effect was observed at 10 µg/ml, so this concentration was used in subsequent cell experiments.

**Fig. 5:**
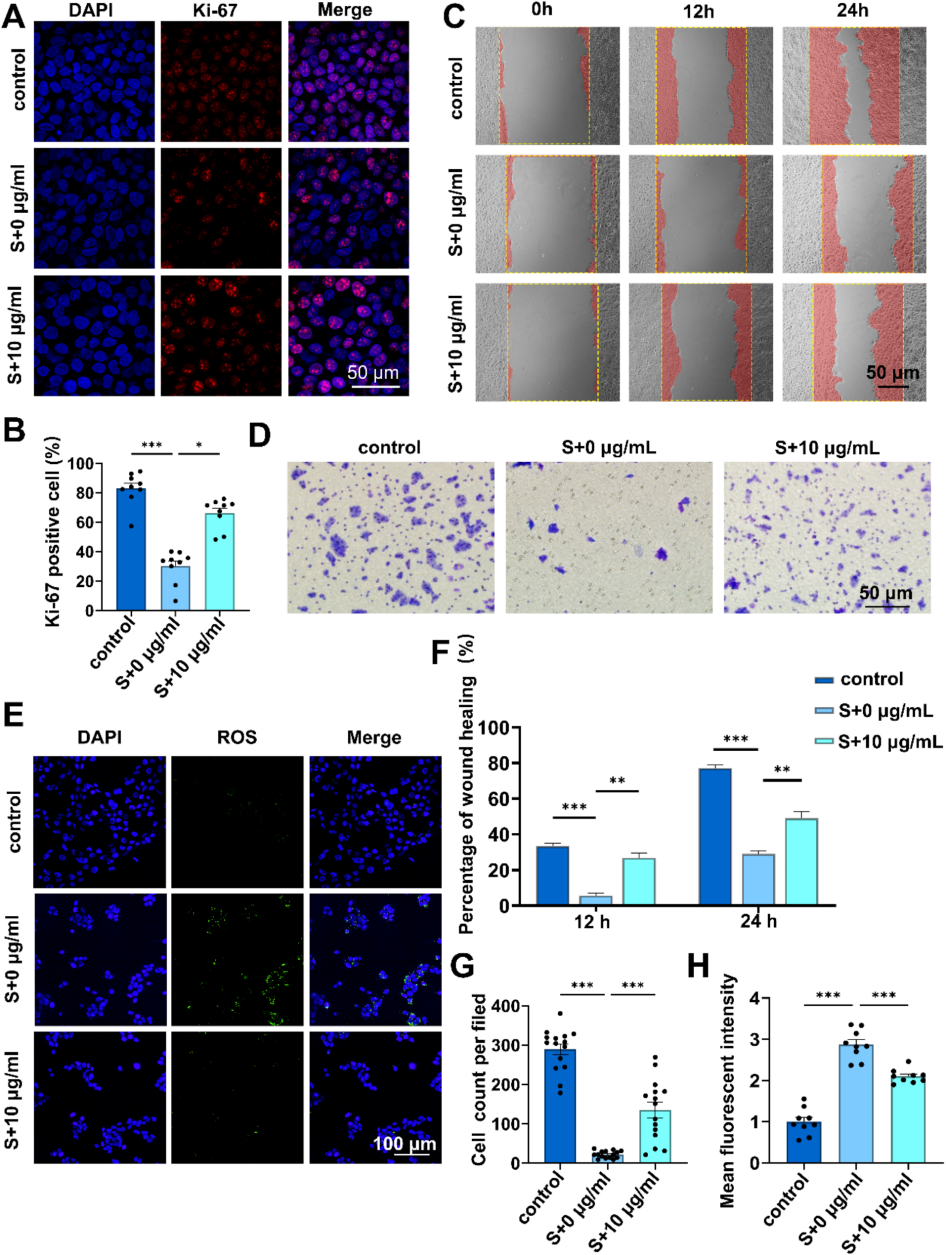
Young fibroblast-derived migrasomes promote senescent HaCaT cells functions. HaCaT cells were co-cultured with or without the young fibroblast-derived migrasomes (10 µg/ml) following treatment with H₂O₂ (600 µmol/L) for 4 h **A**. Representative images of Ki-67 (red) and DAPI (blue) immunofluorescence staining in HaCaT cells **B**. Quantification of Ki-67 immunofluorescence staining in HaCaT cells. Experiments were conducted independently three times. Data are presented as mean ± SD with significance determined by normality and lognormality tests followed by the Kruskal-Wallis test **C**. Representative images of the scratch assay at 12 h and 24 h **D**. Representative images of the transwell migration assay in HaCaT cells **E**. Representative images of ROS (red) and DAPI (blue) staining in HaCaT cells **F**. Quantification of the scratch assay at 12 h and 24 h. Experiments were conducted independently three times. Data were analyzed using one-way ANOVA. Error bars represent the mean ± SEM **G**. Quantification of transwell migration in HaCaT cells. Experiments were performed independently three times. Data are presented as mean ± SEM. Statistical significance was assessed using normality and lognormality tests, followed by the Brown-Forsythe test and Welch’s ANOVA H. Quantification of Ki-67 immunofluorescence staining in HaCaT cells. Experiments were conducted independently three times. Data were analyzed using one-way ANOVA. Error bars represent the mean ± SEM

Furthermore, we examined the impact of young fibroblast-derived migrasomes on the function of senescent HaCaT cells. Results from Ki-67 immunofluorescence staining showed that fibroblast-derived migrasomes enhanced the proliferation activity of senescent HaCaT cells (Fig. 5A and B). Scratch assay (Fig. 5C and F) and transwell assays (Fig. 5D and G) demonstrated that young fibroblast-derived migrasomes significantly increased the migration capacity of senescent HaCaT cells. Considering the association between reactive oxygen species (ROS) and senescence, we examined ROS levels. The results showed that migrasomes reduced the elevated ROS levels in senescent HaCaT cells (Fig. 5E and H). These results indicate that fibroblast-derived migrasomes can alleviate senescence in HaCaT cells.

### Young fibroblast-derived migrasomes promoted wound closure in natural aging mice models

**Fig. 6:**
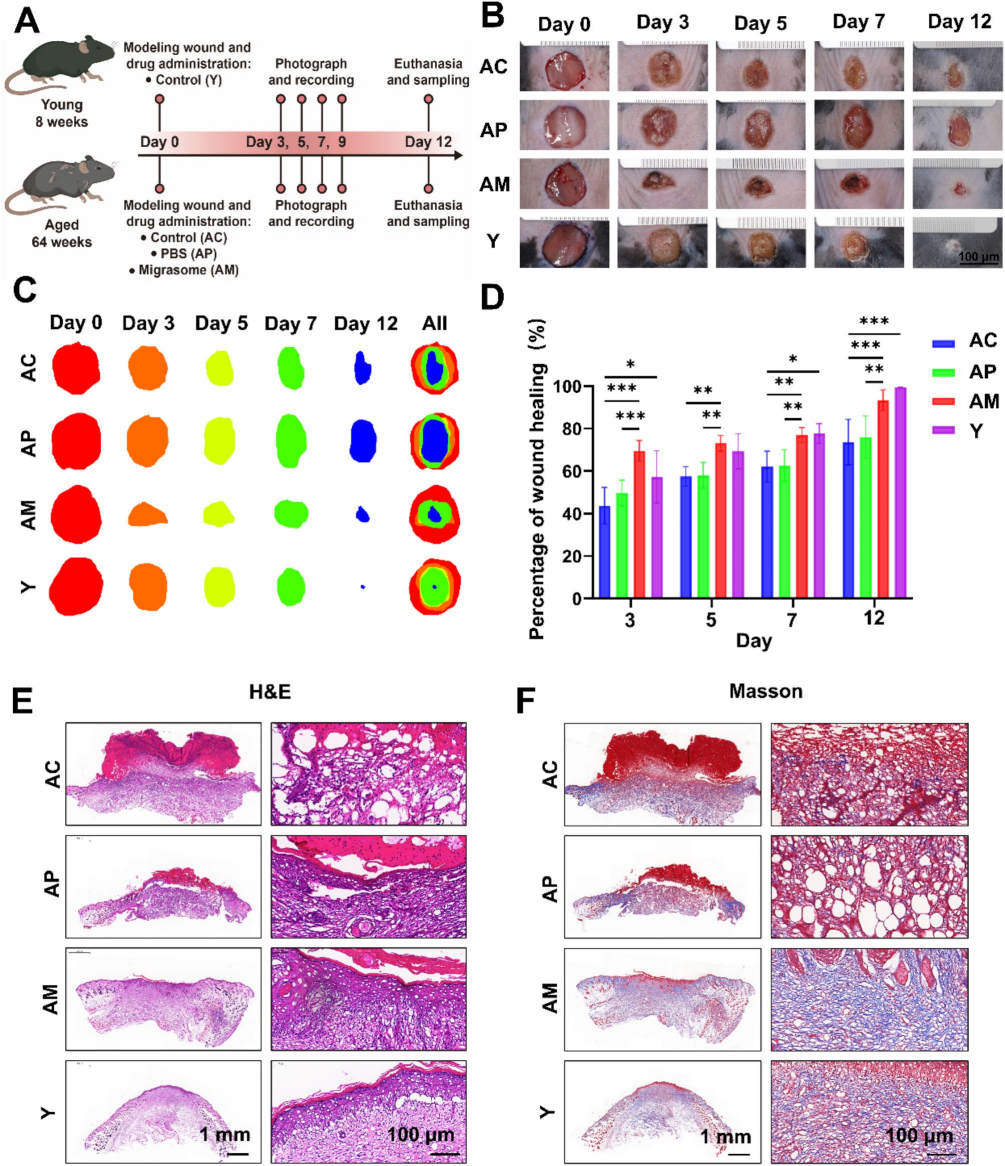
In vivo effects of young fibroblast-derived migrasomes on wound healing in aging mouse skin **A**. Schematic of the experimental design for the mouse wound healing model **B** and **C**. Representative images of skin wounds in young and aging C57BL/6 mice treated with PBS or young fibroblast-derived migrasomes (100 µg) at days 0, 3, 6, 9, and 12 post-surgeries. The mice were randomly assigned to four groups: Young Mouse Group (Y), Aged Mouse Control Group (AC), Aged Mouse PBS-Injected Group (AP), and Aged Mouse Migrasome-Injected Group (AM). The young mouse group consists of 3 mice, and the aging mouse groups consist of 5 mice each.**D**. Quantification of wound area during the healing process. Data are presented as mean ± SD. Young mice (*n* = 3) and aging mice (*n* = 5) were analyzed. Statistical significance was assessed using two-way ANOVA. Error bars represent the mean ± SD **E**. Representative images of skin wounds stained with hematoxylin and eosin (H&E) after 12 days of treatment **F**. Representative images of skin wounds stained with Masson’s trichrome after 12 days of treatment

Considering that senescence impairs the regenerative capacity of damaged tissues [31] and the impact of young fibroblast-derived migrasomes on HaCaT cells, we investigated whether these migrasomes could also promote wound healing in aged skin. We established a full-thickness skin wound model. The mice were randomly assigned to four groups: young mouse group (Y), aged mouse control group (AC), aged mouse PBS-injected group (AP), and aged mouse migrasome-injected group (AM). The AP and AM received subcutaneous injections of 25 µg per site (total four sites) around the wound. The model was photographed and recorded on days 0, 3, 5, 7 and 12, with samples collected on day 12 (Fig. 6A). The observations revealed that the young group healed significantly faster than both the AC and AP groups, highlighting the adverse effects of aging on wound healing. There was no significant difference between AP and AC, but the AM showed significantly faster healing speed than both AC and AP. Interestingly, the healing ability of AM was even better than the young group during the first 7 days, but was surpassed by the young group afterward. This phenomenon might be due to the depletion of migrasomes 7 days after the injury in AM (Fig. 6B, C and D).

HE staining revealed that the AC and AP displayed disorganized re-epithelialization, while the AM exhibited superior re-epithelialization (Fig. 6E). Masson staining indicated that compared with the AC and AP, AM showed increased dermal thickness and collagen fiber formation (Fig. 6F).

**Fig. 7:**
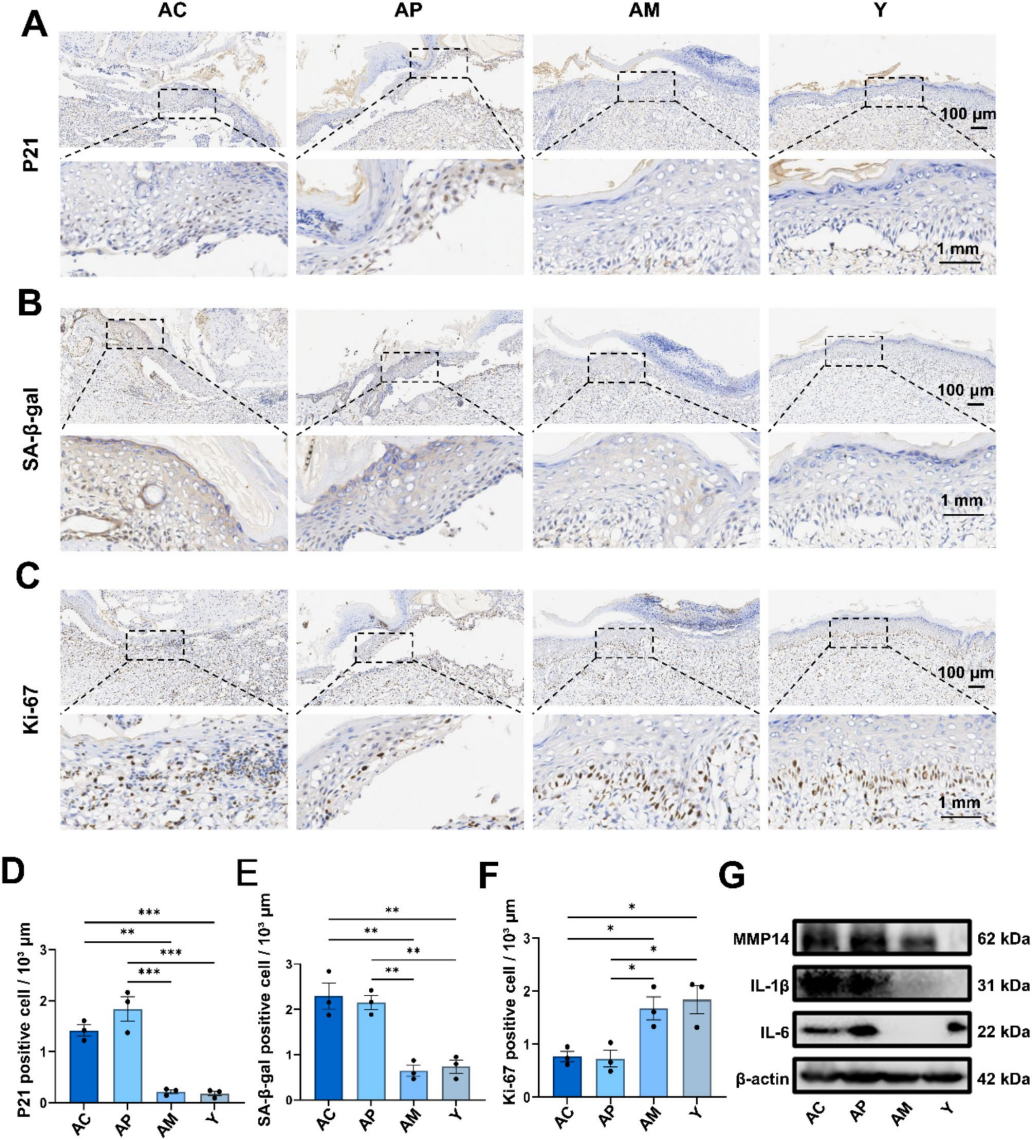
In vivo effects of young fibroblast-derived migrasomes on reducing senescent cells and SASP **A**. Representative immunohistochemical staining images for p21 **B**. Representative immunohistochemical staining images for SA-β-gal **C**. Representative immunohistochemical staining images for Ki-67 **D**. Quantification of immunohistochemical staining images for p21. Experiments were conducted independently three times. Data were analyzed using one-way ANOVA. Error bars represent the mean ± SEM **E**. Quantification of immunohistochemical staining images for SA-β-gal. Experiments were conducted independently three times. Data were analyzed using one-way ANOVA. Error bars represent the mean ± SEM **F**. Quantification of immunohistochemical staining images for Ki-67. Experiments were conducted independently three times. Data were analyzed using one-way ANOVA. Error bars represent the mean ± SEM **G**. Representative Western Blot images showing the expression of IL-1β, IL-6 and MMP14

Previous studies have reported that enhanced wound healing via targeting cellular senescence mainly involves reducing senescent cells and SASP [32]. Therefore, we explored the effects of migrasomes injection on senescent cells and SASP via immunohistochemistry and western blot. Immunohistochemical analysis revealed a significant decrease of p21 protein level in the AM and Y compared to AC and AP (Fig. 7A and D). Similarly, SA-β-gal protein level was notably downregulated in the AM and Y groups relative to the AC and AP (Fig. 7B and E). Conversely, Ki-67 protein level was significantly upregulated in the AM and Y groups compared with the AC and AP groups (Fig. 7C and F). These results demonstrated that injection of migrasomes significantly reduced the number of senescent cells. We also tested pro-inflammatory cytokines IL-1β, IL-6, and MMP-14, which are markers of the SASP [6]. The protein level of IL-1β, IL-6, and MMP-14 was significantly downregulated in the AM and Y groups compared with the AC and AP groups (Fig. 7G), suggesting that migrasomes can alleviate SASP.

## Discussion

ScRNA-seq revealed that fibroblasts possessed the highest level of transcriptional variability during skin aging. We further discovered that the formation ability of migrasomes were negatively related to fibroblast senescence. Additionally, young fibroblast-derived migrasomes significantly alleviate keratinocyte senescence and promote wound healing in aged skin.

Recent literature has reported that fibroblasts’ secretions exhibit pronounced effects in reversing skin aging [33]. Our research demonstrated that young fibroblast-derived migrasomes alleviate senescence in HaCaT cells. However, as fibroblasts senesce, their regulatory functions gradually decline, which may be a key factor in skin aging [34]. Similarly, our results showed that the formation ability of migrasomes gradually declines as fibroblasts senesce. These findings suggested that the decreased ability of migrasomes formation caused by fibroblast senescence may potentially contribute to skin aging.

Migrasome is a newly discovered organelle that originates from retraction fibers at the rear of migrating cells. After their formation, migrasomes are left behind as cells move away. They may either rupture, releasing their contents into the extracellular space, or be internalized by neighboring cells, facilitating the transfer of materials into those cells. Migrasomes are involved in various functions, including mitochondrial quality control, material exchange, and angiogenesis [15, 26, 35]. Our research, through bioinformatics analysis, histological, and cellular studies, validated a negative correlation between fibroblast senescence and migrasomes formation ability. The formation of migrasomes highly depends on cellular migration capacity [15]. However, cellular senescence often accompanies a decline in cell migration and chemotaxis abilities [36]. This may be a plausible reason for the decreased migrasomes formation ability in senescent cells.

Functional deficiencies caused by cellular senescence lead to impaired healing and regenerative potential of aging-associated tissues [37]. The use of senolytics that target and eliminate senescent cells is an emerging approach for anti-aging and the treatment of aging-related diseases [38]. Our results indicate that migrasome can significantly alleviate senescence in HaCaT cells. Additionally, our in vivo results show that migrasomes accelerate wound healing in aged mice and reduce the expression of senescence-related proteins. This suggests that migrasomes may be a potential therapeutic for combating skin aging. However, the specific mechanisms of migrasomes regulating keratinocyte senescence are still unclear and require subsequent research. We propose two mechanisms through which migrasomes may influence cell senescence: one involves autophagy, and the other involves mitochondria. First, migrasomes contain a substantial number of autophagosomes [28], and an increase in autophagic activity has been shown to alleviate cellular senescence [39]. Therefore, migrasomes may regulate autophagy in senescent keratinocytes, thereby slowing or reversing the progression of senescence. Second, migrasomes contain mitochondria [35], which are closely associated with cell senescence. Through their interaction with mitochondria, migrasomes could influence cellular energy metabolism, potentially alleviating cellular senescence. While these mechanisms still need further experimental validation, we believe they provide a promising direction for future studies. Additionally, our study only utilizes naturally aged mouse models. Further studies should include additional aging models, such as photoaging and D-galactose-induced aging models, to comprehensively evaluate the long-term impact of young fibroblast-derived migrasomes on rejuvenating aged skin.

Nanodrug delivery systems have gained significant attention for skin aging repair [40–42]. Migrasomes, as natural nanocarriers, show great therapeutic potential due to their excellent biocompatibility and low immunogenicity. However, several issues still need to be addressed for their application in anti-aging treatments. Migrasomes are susceptible to rupture, leading to leakage of the cargos. Consequently, using migrasomes as carriers for nanodrugs remains challenged by uneven drug release. Advances in nanotechnology provide potential solutions. For example, surface modification can enhance the stability of migrasomes and reduce environmental interference. Additionally, controlled release systems can optimize drug delivery.

## Conclusion

In summary, the present study reveals a significant decline in migrasomes formation ability during the fibroblast senescence and skin aging progress. Additionally, young fibroblast-derived migrasomes rejuvenate senescent keratinocyte and promote wound healing in aged skin. These findings provide novel insights and directions for the development of new therapies targeting skin aging.

## Electronic supplementary material

Below is the link to the electronic supplementary material.

Supplementary Material 1

Supplementary Material 2

## Data Availability

No datasets were generated or analysed during the current study.

## Abbreviations

BSA: Bovine Serum Albumin
CCA: Canonical Correlation Analysis
CDK: Cyclin-Dependent Kinase
DEG: Differentially Expressed Gene
GEO: Gene Expression Omnibus database
H&E: Hematoxylin and Eosin
mIHC: Multiplex Immunohistochemistry
PBS: Phosphate Buffered Saline
PFA: Paraformaldehyde
PMSF: Phenylmethanesulfonylfluoride
RIPA: Radioimmunoprecipitation
ROS: Reactive Oxygen Species
scRNA-seq: Single-cell RNA sequencing
SEM: Scanning electron microscopy
SASP: Senescence-associated secretory phenotype
SA-β-gal: Senescence-Associated-β-Galactosidase
SNN: Shared Nearest Neighbor
TEM: Transmission electron microscopy
t-SNE: t-Distributed Stochastic Neighbor Embedding
WGA: Transmission electron microscopy
