# Supplementary material for: Young fibroblast-derived migrasomes alleviate keratinocyte senescence and enhance wound healing in aged skin
Source: J Nanobiotechnology. 2025 Mar 11;23:200. doi: 10.1186/s12951-025-03293-2 (PMC11895310; doi:10.1186/s12951-025-03293-2)
Supplement: Supplementary file 1 — Supplementary Material 1 [file 12951_2025_3293_MOESM1_ESM.docx]

**Young Fibroblasts-Derived Migrasomes Alleviate Keratinocyte Senescence and Enhance Wound Healing in Aged Skin**

**Hanlin Tu^a, 1^, Yingliang Shi^a, 1^, Yi Guo^a^, Zhongyang Zou^a^, Yuyan He^a^, Jing Zhou^a^, Sangang He^a, b,^ *, Guoliang Sa^a, b,^ ***

^a^ State Key Laboratory of Oral & Maxillofacial Reconstruction and Regeneration, Key Laboratory of Oral Biomedicine Ministry of Education, Hubei Key Laboratory of Stomatology, School & Hospital of Stomatology, Wuhan University, Wuhan 430079, China.

^b^ Department of Oral and Maxillofacial surgery, School and Hospital of Stomatology, Wuhan University, Wuhan, 430079

^1^ These authors contribute equally to this work.

***** Corresponding author: Sangang He, Guoliang Sa.

E-mail addresses: sangang@whu.edu.cn (S. H); guoliang@whu.edu.cn (G. Sa).

**Materials and methods**

**Transmission Electron Microscopy (TEM)**

Ultra-thin sections of skin samples and negatively stained migrasomes were observed using TEM. Skin samples were trimmed into 1 mm × 1 mm × 3 mm blocks with ophthalmic scissors and fixed in electron microscopy fixative (G1102, Servicebio, China). Samples were then fixed in 1% osmium tetroxide (18456, Ted Pella Inc, USA) at room temperature for 2 hours, dehydrated through a graded series of ethanol (30%, 50%, 70%, 80%, 95%, 100%, 100%), and embedded in acetone resin. Ultra-thin sections of the embedded specimens were prepared and placed on 150-mesh copper grids (WFHM-150, Servicebio, China) for observation with a transmission electron microscope (HT7700, HITACHI, Japan). Purified migrasomes were placed on 200-mesh copper grids for 5-10 minutes, excess liquid was absorbed with filter paper, stained with 2% uranyl acetate, and observed using the transmission electron microscope (HT7700, HITACHI, Japan).

**Scanning Electron Microscopy (SEM)**

After cell cultured on the cell slides, the medium was discarded, and samples were gently washed with PBS, then fixed in electron microscopy fixative at room temperature for 2 hours. This was followed by fixation in 1% osmium tetroxide for 2 hours. Samples were then dehydrated through a graded ethanol series (30%, 50%, 70%, 80%, 95%, 100%, 100%) for 15 minutes each, and subsequently treated with isoamyl acetate for 15 minutes. The samples were dried in a critical point dryer, gold spraying for about 30 seconds using an ion sputtering instrument (MC1000, Hitachi, Japan), and observed with a scanning electron microscope (SU8100, Hitachi, Japan).

**Induction of HaCaT and BJ cells senescence**

HaCaT cells were treated with 600 μmol/L H_2_O_2_ (H792072, Macklin, China) (diluted in serum-free medium) for 4 hours in the incubator to induce senescence. Cells were then cultured in fresh complete medium, and after 48 hours, relevant indicators were assessed for subsequent experiments. BJ cells were treated with 200 μmol/L H_2_O_2_ (diluted in serum-free medium) for 12 hours, and after 48 hours, and the relevant indicators were evaluated for subsequent experiments.

**Senescence-Associated-β-Galactosidase (SA-β-gal) Staining**

Cells were fixed with fixative for 15 minutes and then stained with freshly prepared staining solution at 37°C for 24 hours, according to the SA-β-gal assay kit manual (C0602, Beyotime, China). Result was observed and then photographed through microscope (IX83, Olympus, Japan).

**Migrasomes internalization assay**

Migrasomes were stained with 5 μg/ml red wheat germ agglutinin (WGA) (W7024, Thermo Scientific, USA) for 15 minutes label migrasomes. Then, the labeled migrasomes were added to senescent HaCaT cells and co-cultured for 4 hours. HaCaT cells were washed with PBS and fixed in 4% PFA for 15 minutes, and stained with phalloidin. After washing with PBS three times, stain the nuclei with DAPI and observe using a confocal microscope (FV1200, Olympus, Japan).

**Western blotting**

Cells and migrasomes were lysed using Radioimmunoprecipitation (RIPA) lysis buffer (P0013B, Beyotime, China) containing 1% Phenylmethanesulfonylfluoride (PMSF) (ST506, Beyotime, China). Protein concentration was measured with the BCA Protein Assay Kit, and western blotting was performed according to previously reported protocols.

For tissue samples, after being washed with PBS, the samples were placed into a centrifuge tube. Subsequently, metal grinding beads (F6621, Beyotime, China) and 1 mL of RIPA buffer containing 1% PMSF were added per 0.1 g of tissue. The tissue was then homogenized using a tissue homogenizer (OSE-TH-02, TIANGEN, China). Following homogenization, the resultant liquid was transferred to a fresh tube, subjected to sonication, and incubated on ice for 30 minutes. Western blotting was performed according to previously reported protocols.

**Immunofluorescence staining**

Cells were first fixed at room temperature with 4% PFA for 15 minutes. The cells were then washed three times with PBS. Permeabilization was performed with 0.5% Triton X-100 (P0096, Beyotime, China) at room temperature for 10 minutes, followed by three washes with PBS. Then cell was blocked with 2% bovine serum albumin (BSA) (ST023, Beyotime, China) at 37°C for 1 hour, followed by overnight incubation with the primary antibody at 4°C. And goat anti-rabbit or anti-mouse conjugated to Alexa Fluor 594 or 488 was incubated at room temperature for 1 hour. The cells were then washed three times with PBS and stained with DAPI.

As for tissue immunofluorescence staining, after deparaffinization, tissue sections were placed in 1×EDTA at 95 ℃ for antigen repair. The subsequent steps are the same as described above.

**Transwell assays**

We conducted a transwell assay using 24-well transwell inserts with an 8 μm pore size filter (14341, Labselect, China) and 24-well culture plates. Cells were suspended in low-serum medium (containing 1% FBS) and seeded into the upper chamber at a density of 1 × 10^5^ cells per well. The lower chamber was filled with 500 μL of complete medium (containing 10% FBS). After 24 hours, cells adhering to the upper surface of the filter membrane were removed with a cotton swab, and the cells on the underside of the filter membrane (migrated cells) were stained with crystal violet. Each group was tested in triplicate.

**Scratch assay**

Cells at a density of 3 × 10^5^ cells per well were seeded and incubated in 12-well plates at 37 °C. After cell attachment, a scratch was made in the monolayer cells using a 200 μL pipette tip. The wells were then washed with PBS to remove floating cells. Images of HaCaT cells were captured at 0 h, 12 h, and 24 h. The migration area rate was determined as the ratio of the closed area to the initial wound area. Migration area (%) = (A1 – A0) / A1 × 100, where A1 represents the initial wound area and A0 represents the remaining wound area analyzed by ImageJ software. Each group was tested in triplicate.

**Reactive oxygen species (ROS) detection**

The intracellular production of reactive oxygen species (ROS) in HaCaT cells was quantified utilizing the ROS kit (S0033S, Beyotime, China). Specifically, HaCaT cells from each group (n = 3 per group) were plated in dishes containing the appropriate medium. Subsequent to H_2_O_2_ stimulation, the cells were incubated with the migration medium for 24 hours. Thereafter, the cells were incubated with the ROS kit at 37°C for 30 minutes. Following this incubation, the cells were meticulously washed with serum-free DMEM and subsequently observed under a confocal microscope.

**Immunohistochemistry**

Skin tissues were fixed in 4% PFA for 24 hours and embedded in paraffin. Following dewaxing, 5 μm thick sections centered on the wound were prepared for HE and Masson staining. Antigen retrieval was conducted with 1× EDTA buffer (P005, Beyoime, China) at 95 °C for 15 minutes. Subsequent procedures followed standard immunohistochemical methods.

**Antibodies used for Western blot,** **IHC or IF**

| **antibody** | **host** | **source** | **application** |
| --- | --- | --- | --- |
| P21 | Rabbit | Proteintech (10355-1-AP) | WB：1:1500  IHC: 1:50 |
| P16-INK4A | Rabbit | Proteintech (10883-1-AP) | WB: 1:1000  IHC: 1:50 |
| Beta Galactosidase | Rabbit | Proteintech (15518-1-AP1) | IHC: 1:50 |
| PIGK | Rabbit | Abcam (ab201693 | WB: 1:1000 |
| EOGT | Rabbit | Abcam (ab190693) | WB: 1:1000 |
| TSPAN4 | Rabbit | Biorbyt (orb41336) | WB: 1:1000  IHC: 1:50 |
| GAPHD | Mouse | Proteintech (60004-1-AP) | WB: 1:10000 |
| Beta Actin | Mouse | Proteintech (66009-1-AP) | WB: 1:10000 |
| Vimentin | Rabbit | Bioss (BS-0756R) | IHC: 1:50  IF: 1:50 |
| Ki-67 | Rabbit | Proteintech (27309-1-AP) | IHC: 1:50  IF: 1:50 |
| IL-6 | Rabbit | Proteintech (21865-1-AP) | WB: 1:1000 |
| IL-1β | Rabbit | Proteintech (16806-1-AP) | WB: 1:1000 |
| MMP-14 | Mouse | R&D System (MAB9181) | WB: 1:1000 |

**
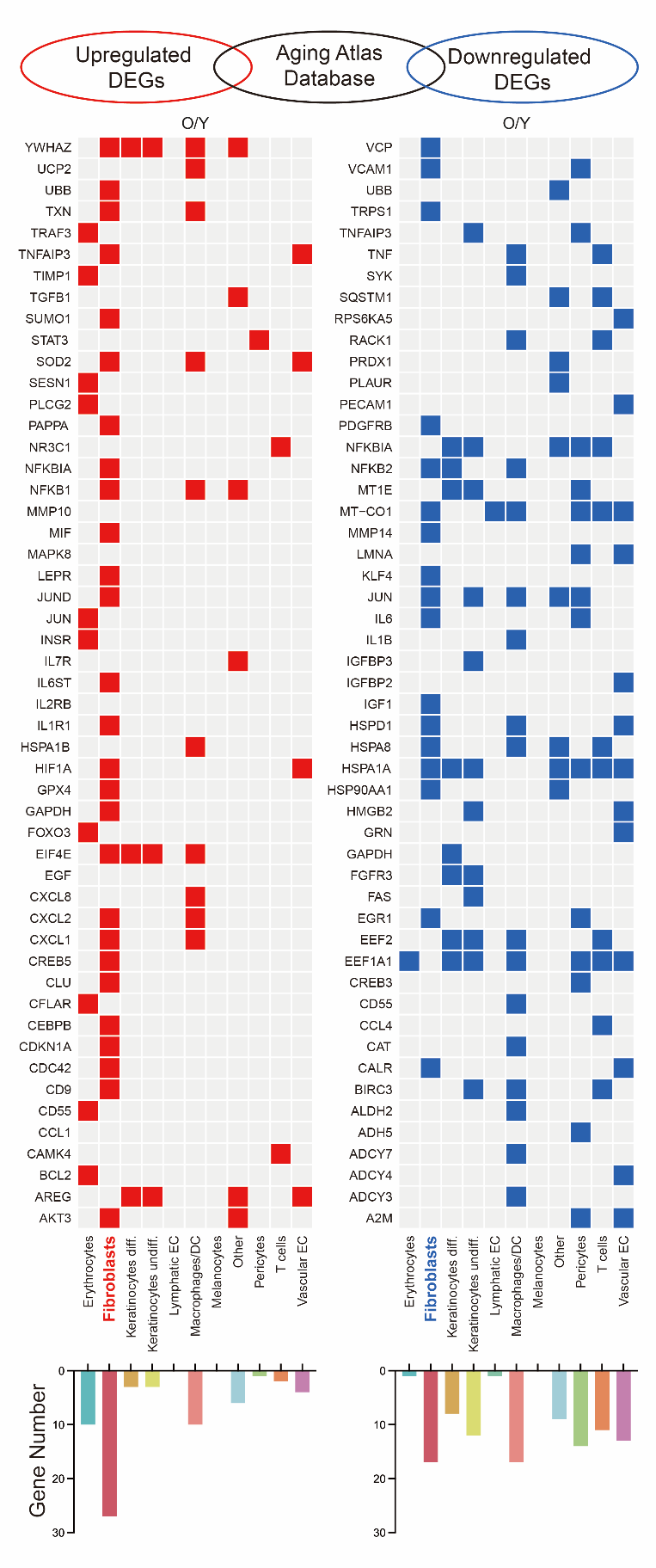
Figures and Figure legends**

**Supplementary Figure 1.** Heatmap shows genes shared between aging-related genes in the Aging Atlas database and DEGs for each skin cell type. Only the ensembles of
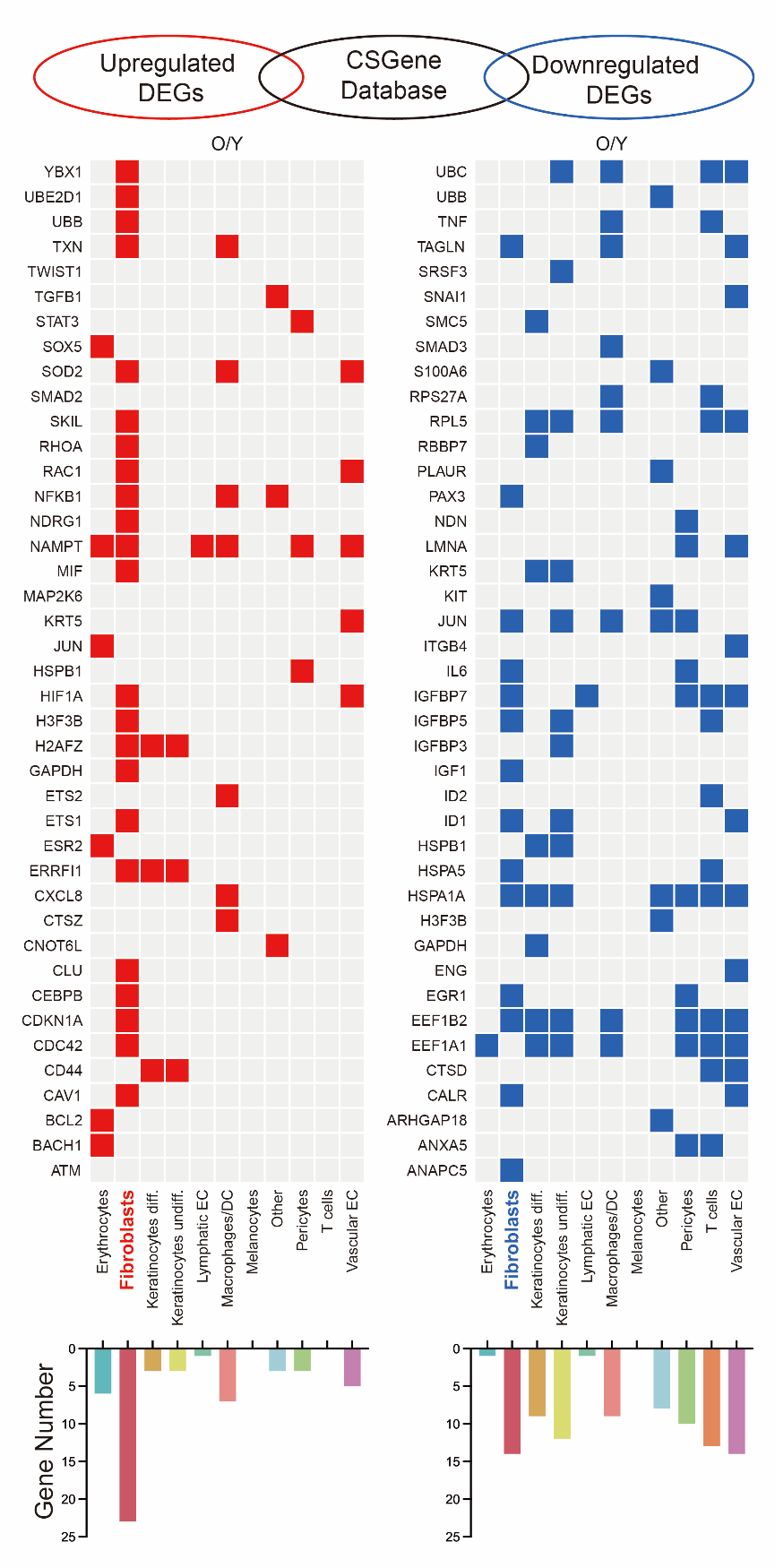
shared genes are shown, and the number statistics are shown below the heatmap.

**Supplementary Figure 2.** Heatmap shows genes shared between genes in the CSGene database and DEGs for each skin cell type. Only the ensembles of shared genes are shown, and the number statistics are shown below the heatmap.


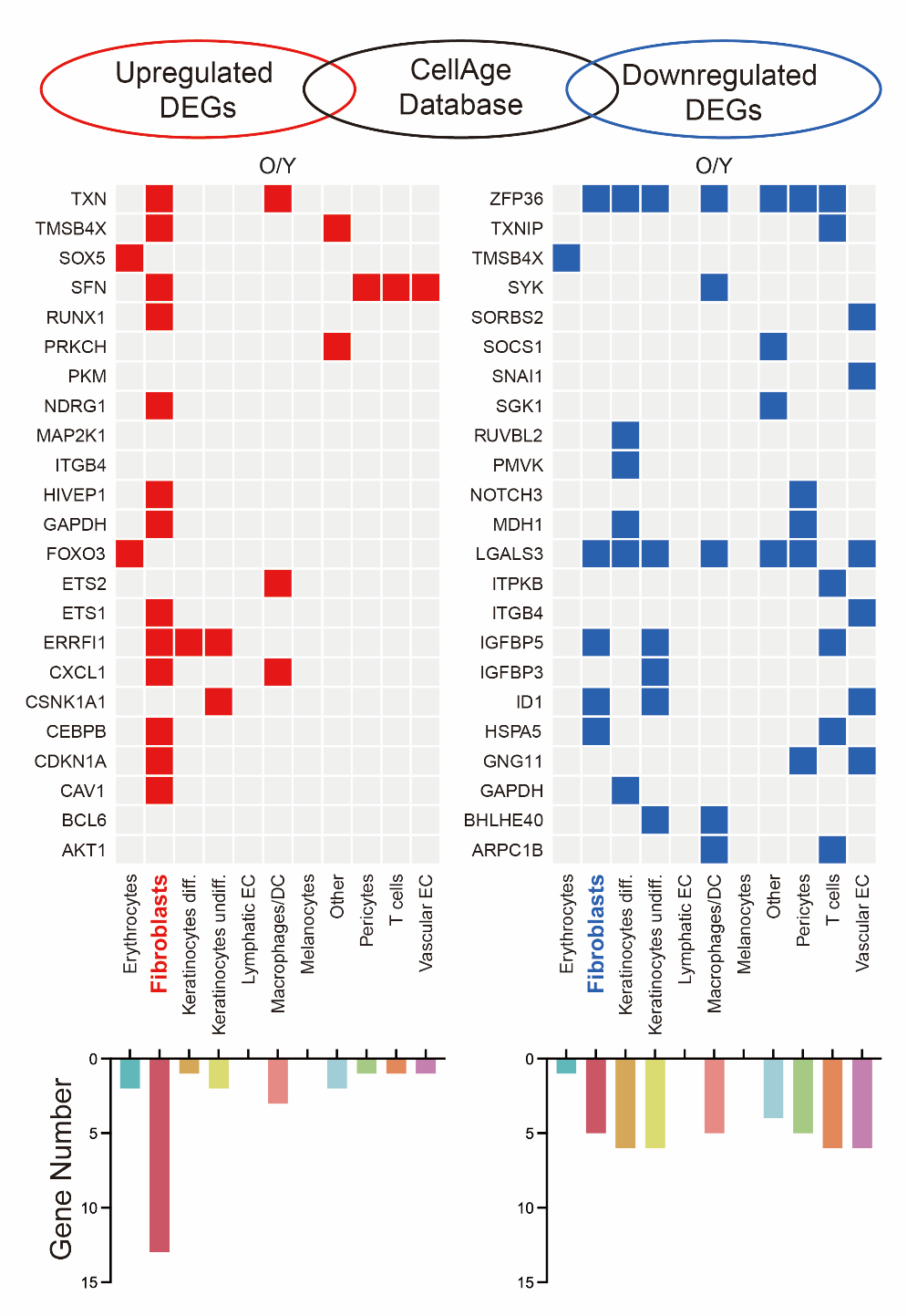
**Supplementary Figure 3.** Heatmap shows genes shared between genes in the CellAge database and DEGs for each skin cell type. Only the ensembles of shared genes are shown, and the number statistics are shown below the heatmap.


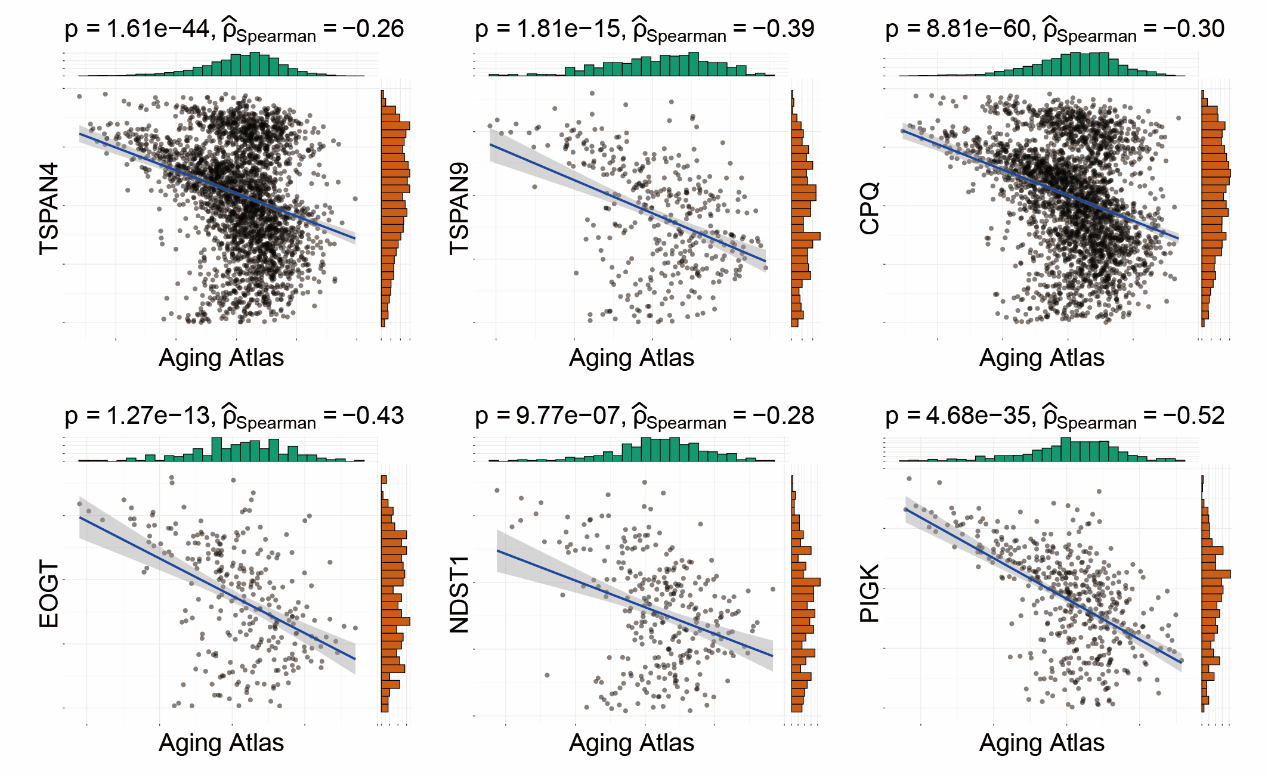


**Supplementary Figure 4.** Scatterplots show the correlation between the six migrasome-related genes (TSPAN4, TSPAN9, CPQ, EOGT, NDST1, and PIGK) and aging-related genes in the Aging Atlas database respectively (see Methods for details). The trend lines represent the overall trend of all the scatter points in the corresponding plots, and the corresponding p-value and Spearman's correlation coefficient are shown at the top of the plots.


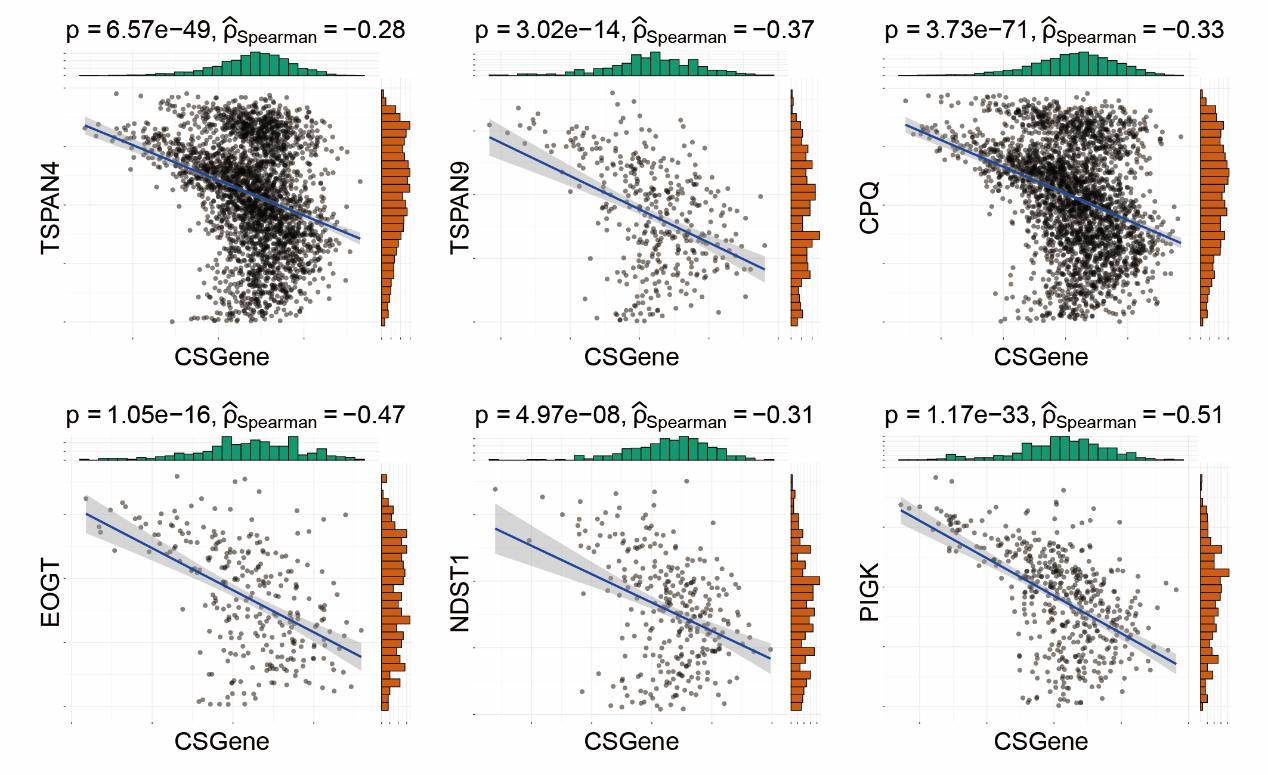


**Supplementary Figure 5.** Scatterplots show the correlation between the six migrasome-related genes (TSPAN4, TSPAN9, CPQ, EOGT, NDST1, and PIGK) and aging-related genes in the CSGene database respectively (see Methods for details). The trend lines represent the overall trend of all the scatter points in the corresponding plots, and the corresponding p-value and Spearman's correlation coefficient are shown at the top of the plots


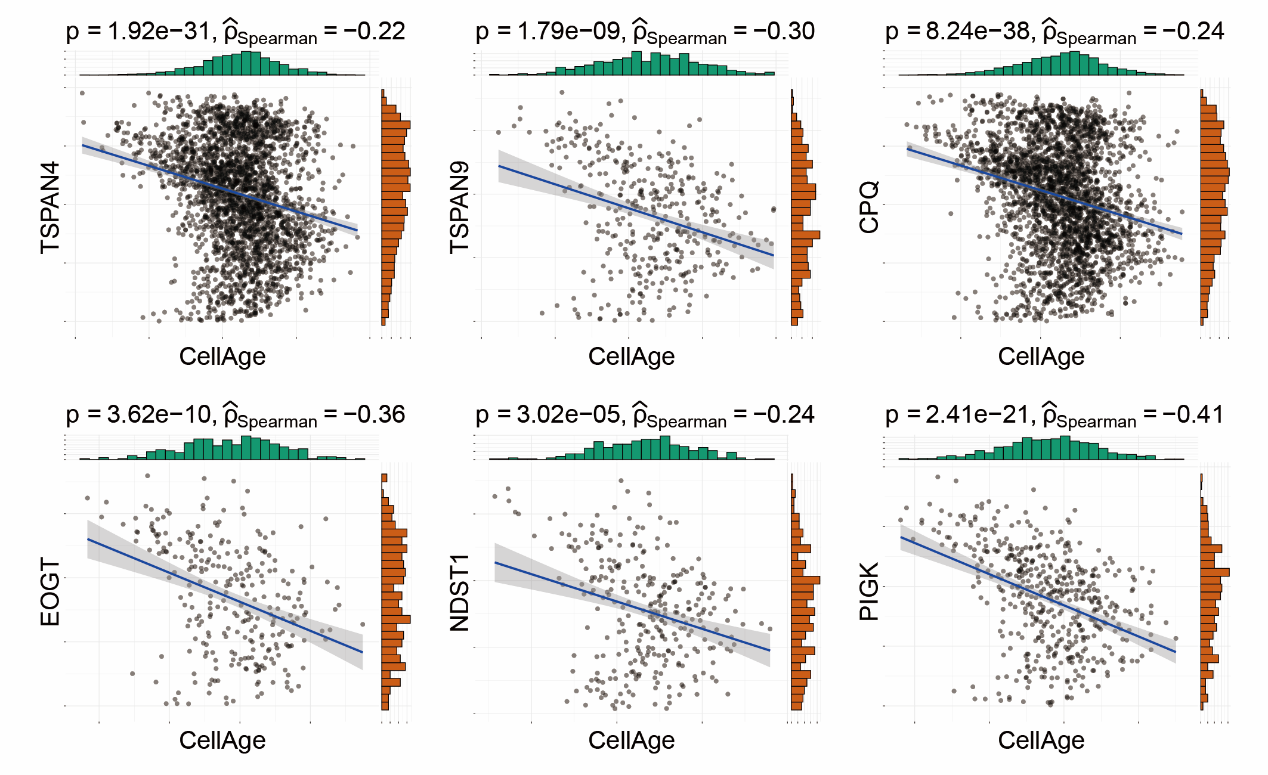


**Supplentary Figure 6**. Scatterplots show the correlation between the six migrasome-related genes (TSPAN4, TSPAN9, CPQ, EOGT, NDST1, and PIGK) and aging-related genes in the CellAge database respectively (see Methods for details). The trend lines represent the overall trend of all the scatter points in the corresponding plots, and the corresponding p-value and Spearman's correlation coefficient are shown at the top of the plots.


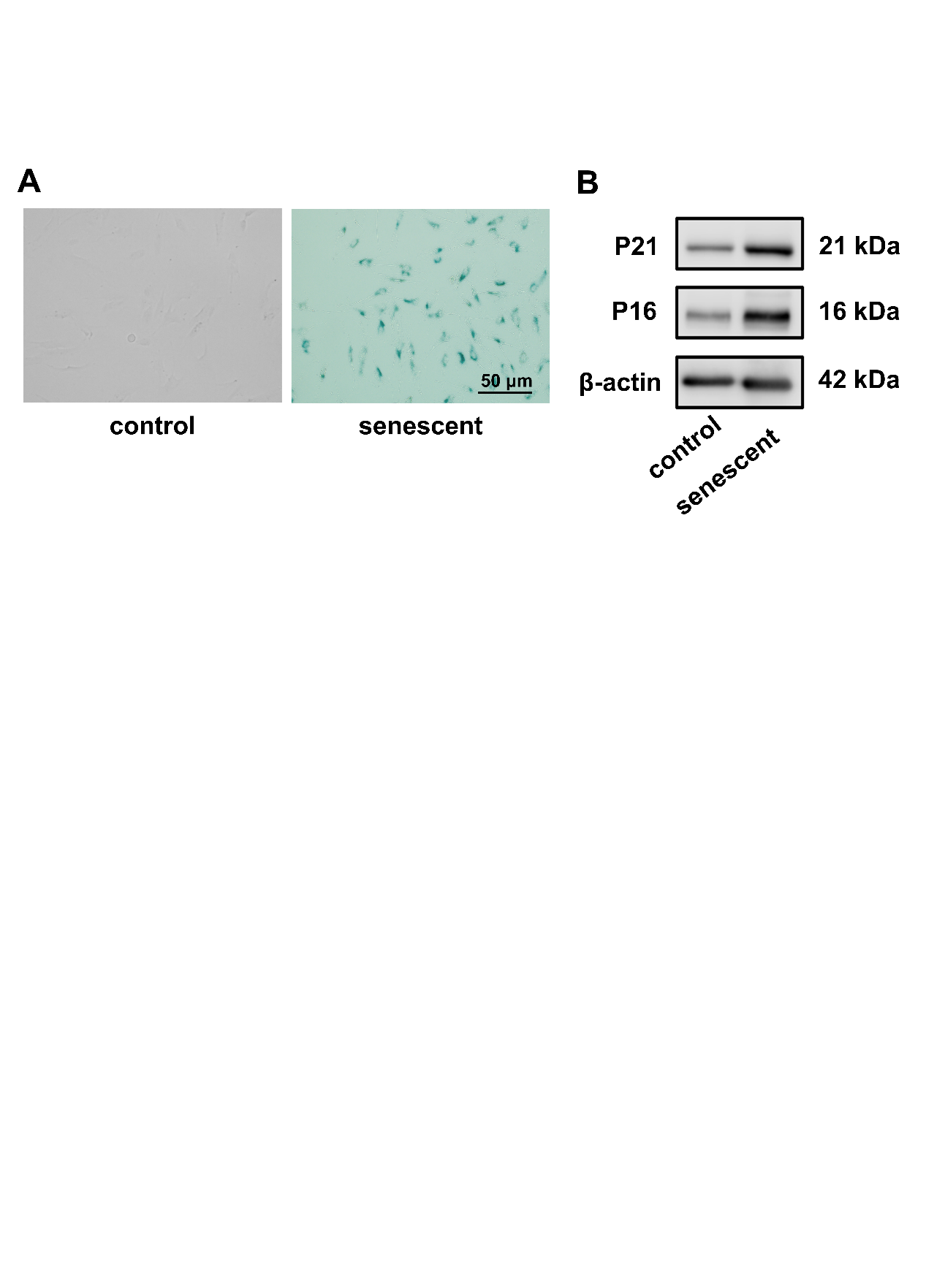


**Supplementary Figure 7.** H₂O₂ induced BJ cell senescence.

A. Representative images of SA-β-gal staining in BJ cells treated with 200 μmol/L H₂O₂.

B. Representative Western Blot images showing the expression of senescence markers P16 and P21 in BJ cells exposed to H₂O₂.


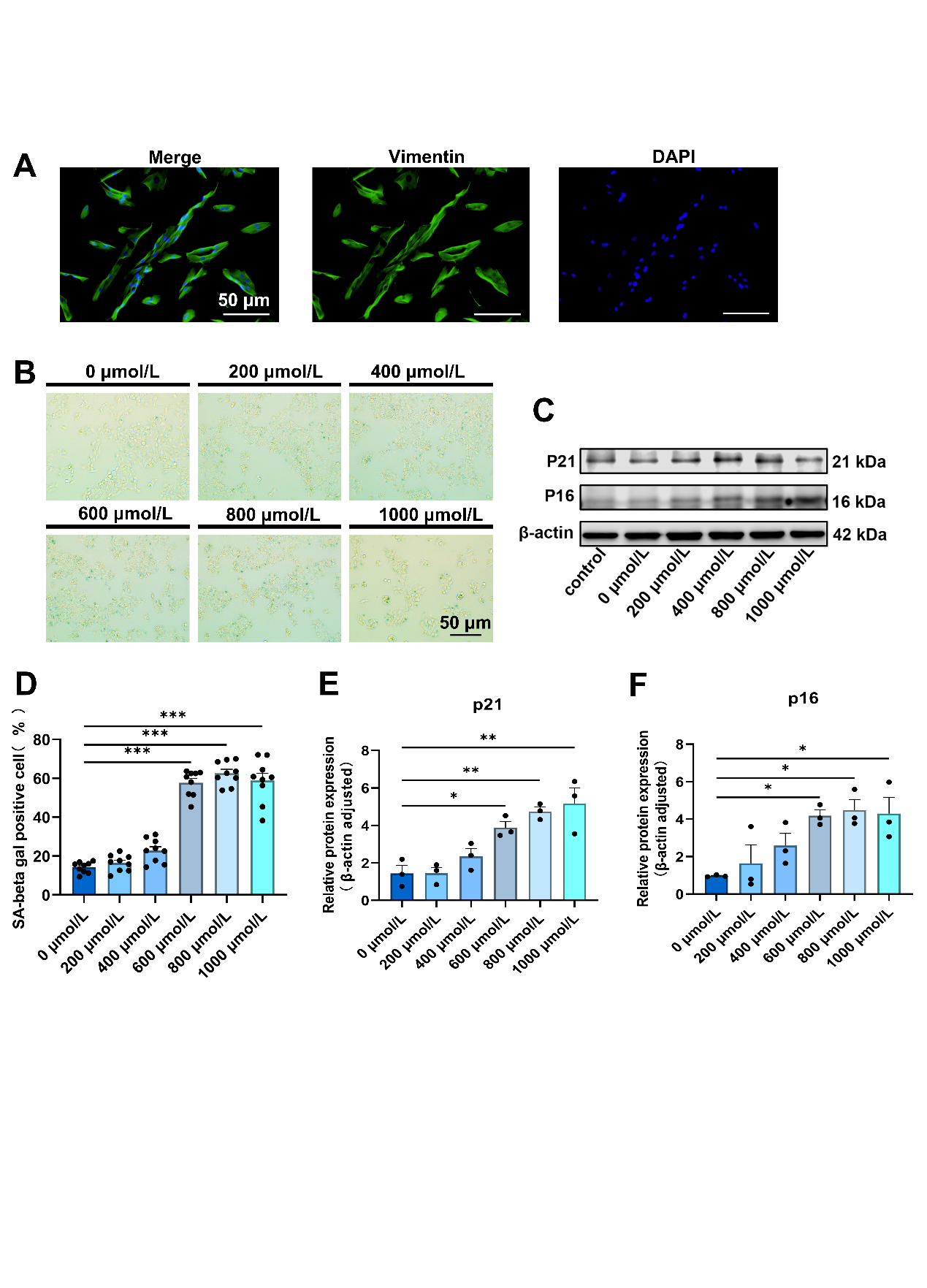


**Supplementary Figure 8.** H₂O₂ induced HaCaT cell senescence.

A. Vimentin immunofluorescence staining of extracted fibroblasts.

B. Representative images of SA-β-gal staining in HaCaT cells treated with different concentrations of H₂O₂.

C. Representative Western Blot images showing the expression of senescence markers P16 and P21 in HaCaT cells exposed to different concentrations of H₂O₂.

D. Quantification of SA β-gal positive cells in HaCaT cells treated with different concentrations of H₂O₂. Experiments were repeated independently three times. Data were collected by randomly photographing 3 fields per group. Statistical analysis was performed using one-way ANOVA. Error bars indicate the mean ± SEM.

E and F. Quantitative Western Blot analysis of senescence markers P16 and P21 in HaCaT cells exposed to H₂O₂ for 48 hours. Experiments were repeated independently three times. Data were analyzed using one-way ANOVA. Error bars indicate the mean ± SEM.
